# Supplementary material for: Subjective ratings of emotive stimuli predict the impact of the COVID-19 quarantine on affective states
Source: PLoS One. 2020 Aug 13;15(8):e0237631. doi: 10.1371/journal.pone.0237631 (PMC7425917; doi:10.1371/journal.pone.0237631)
Supplement: S1 File — (PDF) [file pone.0237631.s001.pdf]

Subjective ratings of emotive stimuli predict the impact of the COVID-19 quarantine on affective states

Héctor López-Carral, Klaudia Grechuta, Paul F.M.J. Verschure

Image Selection

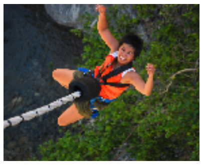
Bungee jumping 3

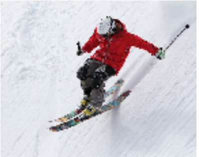
Cold 6

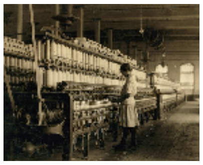
Child labor 4

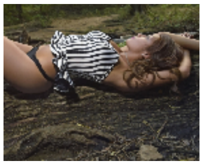
Nude woman 11

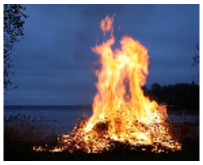
Fire 4

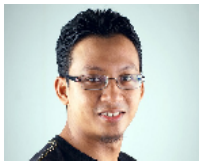
Neutral face 2

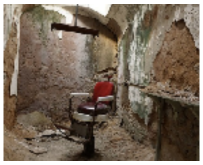
Jail 3

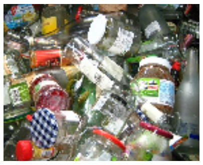
Garbage dump 7

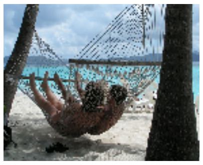
Beach 6

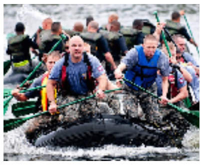
Exercise 3

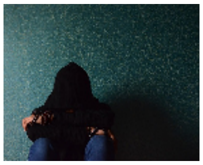
Sad pose 4

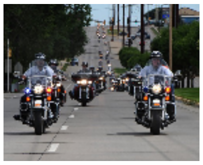
Police 3

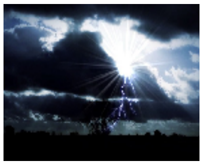
Thunderstorm 2

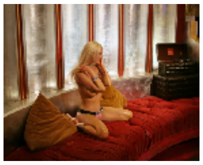
Nude woman 22

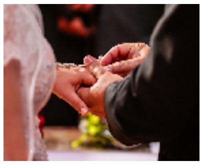
Wedding 9

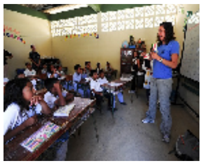
School 1

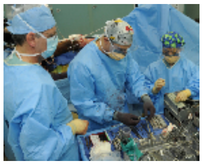
Surgery 3

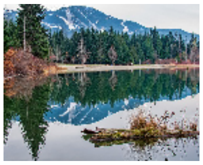
Lake 13

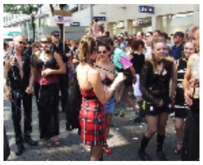
BDSM 1

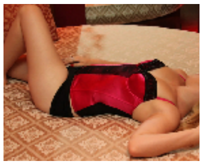
Nude woman 14

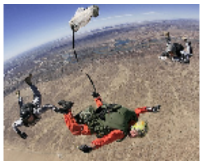
Skydiving 5

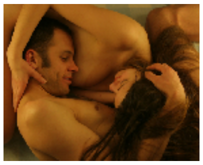
Nude couple 5

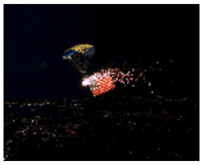
Parasailing 4

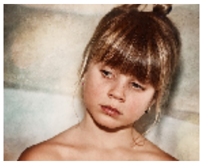
Sad face 5

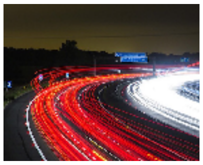
Traffic 1

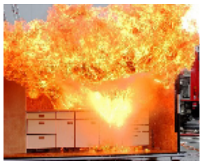
Explosion 6

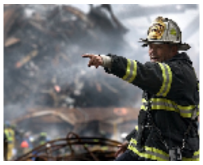
Fireman 1

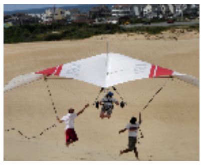
Hang gliding 2

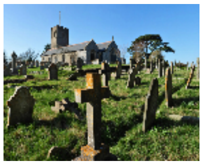
Graveyard 4

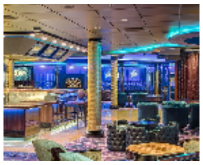
Bar 2
