# Supplementary material for: Subjective ratings of emotive stimuli predict the impact of the COVID-19 quarantine on affective states
Source: PLoS One. 2020 Aug 13;15(8):e0237631. doi: 10.1371/journal.pone.0237631 (PMC7425917; doi:10.1371/journal.pone.0237631)
Supplement: S2 File — (PDF) [file pone.0237631.s002.pdf]

Subjective ratings of emotive stimuli predict the impact of the COVID-19 quarantine on affective states

Héctor López-Carral, Klaudia Grechuta, Paul F.M.J. Verschure

COVID-19 Questionnaire

Question	Input	Options
Are you currently employed?	Multiple choice	Yes; No
Are you currently in quarantine due to COVID-19?	Multiple choice	Yes / No
How many times have you left the house in the last 14 days?	Number	—
Can you work from home?	Multiple choice	Yes; No
(If participants answered Yes in the previous question) Do you enjoy it?	Slider	Range from "not at all" to "very much"
Who do you live with?	Multiple choice	Parents; Boyfriend/Girlfriend; Husband/Wife; Husband/Wife and kid(s); Peers/Flat mates; Alone
Do you like the place where you live?	Slider	Range from "not at all" to "very much"
Would you consider yourself a hypochondriac, that is, someone who lives with the fear that they have a serious, but undiagnosed medical condition, even though diagnostic tests show there is nothing wrong?	Slider	Range from "not at all" to "very much"
Has anyone that you care about been infected with COVID-19?	Multiple choice	Yes, my relative; Yes, myself; Yes, a friend; No
Have you experienced a feeling of fear during the period of the quarantine?	Slider	Range from "not at all" to "very much"
Have you experienced a feeling of panic during the period of the quarantine?	Slider	Range from "not at all" to "very much"
How much do you miss the "normal" pre-quarantine life?	Slider	Range from "not at all" to "very much"
Do you enjoy the quarantine and isolation?	Slider	Range from "not at all" to "very much"
Please, feel free to write your comments or thoughts regarding the social and economic aspects related to the quarantine period: (optional)	Text field	—
